# Expanding the Phenotypic and Genetic Spectrum of Neuromuscular Diseases Caused by *DYNC1H1* Mutations

**DOI:** 10.3389/fneur.2022.943324

**Published:** 2022-07-11

**Authors:** Jia-Tong Li, Si-Qi Dong, Dong-Qing Zhu, Wen-Bo Yang, Ting Qian, Xiao-Ni Liu, Xiang-Jun Chen

**Affiliations:** ^1^Department of Neurology, Huashan Hospital and Institute of Neurology, Fudan University, Shanghai, China; ^2^National Center for Neurological Disorders, Shanghai, China; ^3^Human Phenome Institute, Fudan University, Shanghai, China

**Keywords:** neuromuscular disease, spinal muscular atrophy, Charcot-Marie-Tooth (CMT) disease, *DYNC1H1*, genotype-phenotype correlation

## Abstract

**Objectives:**

Spinal muscular atrophy with lower extremity predominance 1 (SMALED1) and Charcot–Marie-Tooth diseasetype 2O (CMT2O) are two kinds of hereditary neuromuscular diseases caused by *DYNC1H1* mutations. In this study, we reported two patients with SMALED1 caused by *DYNC1H1* mutations. The genotype–phenotype correlations were further analyzed by systematically reviewing previous relevant publications.

**Materials and Methods:**

Two patients' with SMALED1 and their parents' clinical data were collected, and detailed clinical examinations were performed. WES was then applied, which was confirmed by Sanger sequencing. PubMed, Web of Science, CNKI, and Wanfang Data were searched, and all publications that met the inclusion criteria were carefully screened. Any individual patient without a detailed description of clinical phenotypes was excluded.

**Results:**

The two patients manifested delayed motor milestones and muscle wasting of both lower extremities. The diagnosis was further confirmed as SMALED1. Genetic testing revealed heterozygous *DYNC1H1* mutations c.1792C>T and c.790C>G; the latter is a novel dominant mutation. Genotype–phenotype analysis of *DYNC1H1* variants and neuromuscular diseases revealed that mutations in the DYN1 region of DYNC1H1 protein were associated with a more severe phenotype, more complicated symptoms, and more CNS involvement than the DHC_N1 region.

**Conclusion:**

Our study potentially expanded the knowledge of the phenotypic and genetic spectrum of neuromuscular diseases caused by *DYNC1H1* mutations. The genotype–phenotype correlation may reflect the pathogenesis underlying the dyneinopathy caused by *DYNC1H1* mutations.

## Introduction

Located on 14q32.31, the *DYNC1H1* gene encodes dynein cytoplasmic 1 heavy chain 1 (DYNC1H1), which is the core structure of cytoplasmic dynein. Dynein is a large (~1.5 MDa) motor protein complex responsible for retrograde axonal transport in all eukaryotic cells (1). DYNC1H1 plays an essential role in ATPase-dependent movement along the microtubule and recruitment of other dynein subunits (2). Mutations in *DYNC1H1* can lead to various developmental and degenerative diseases of the nervous system, which are nominated as a disease spectrum of “dyneinopathy” characterized by locomotor and motor system deficits, sensory system defects, and (or) brain morphology and function abnormalities (3).

Spinal muscular atrophy with lower extremity predominance 1 (SMALED1, OMIM: 158600) and Charcot–Marie-tooth type 2O (CMT2O, OMIM: 614228) have both been reported as hereditary neuromuscular diseases caused by *DYNC1H1* variants (4–6). Spinal muscular atrophy (SMA) is caused by the impairment of motor neurons in the spinal cord. SMALED1 is an autosomal dominant hereditary type of SMA, characterized by non-length-dependent weakness restricted or predominant in the lower limbs (4, 7, 8). Charcot–Marie-tooth (CMT) disease, also known as hereditary motor and sensory neuropathy (HMSN), is genetically heterogeneous and clinically characterized by progressive distal muscle weakness and wasting, sometimes accompanied by sensory abnormalities. CMT2O was first reported by Weedon et al. in a large four-generation family and the patients presented with delayed motor milestones, abnormal gait, and slowly progressive distal lower limb atrophy and weakness accompanied by *pes cavus* deformity (6).

Although numerous cases have been reported worldwide, the analysis of the genotype–phenotype correlations between *DYNC1H1* mutations and neuromuscular diseases, including SMALED1 and CMT2O, is still scarce. Here, we first reported *DYNC1H1* gene c.1792C>T (p.R598C) and c.790C>G (p.R264G) *de novo* heterozygous mutations in two sporadic SMALED1 cases. Next, we reviewed previous publications and summarized the phenotypic and genetic characteristics of neuromuscular diseases caused by *DYNC1H1* mutations. Furthermore, we focused on the genotype–phenotype correlations of *DYNC1H1* variants, which may provide insights into unraveling the mechanism of the clinical heterogeneity of “dyneinopathy.”

## Methods

### Ethics

The study was approved by the Ethics Committee of Huashan Hospital, Fudan University. The patients and their family members all provided informed consent according to the Declaration of Helsinki.

### Whole-Exome Sequencing (WES) and Candidate Variant Screening

Genomic DNA was extracted from EDTA-treated peripheral blood using a DNA extraction kit (Qiagen, Hilden, Germany). The purity and quality of DNA were examined by 1% agarose electrophoresis and Qubit, respectively. The DNA was then sequenced by WES. All exons of the two probands were sequenced by the HiSeq4000 Illumina Genome Analyzer II platform (Illumina, San Diego, CA). The variants were analyzed according to the previous reference (9): (1) automatically including variants with minor allele frequencies ≤ 0.01 in further analysis by sifting of variants against the Human Genome Mutation Database (HGMD, http://www.hgmd.cf.ac.uk/ac/index.php), the NCBI SNP database (https://www.ncbi.nlm.nih.gov/snp), and the Exome Variant Server (10); (2) filtering those variants which do not code protein and do not alter splice sites as predicted by BDGP (https://www.fruitfly.org/seq_tools/splice.html); (3) ignoring synonymous variants; and (4) combining clinical manifestations and genetic characteristics to screen for associated variants. The detected *DYNC1H1* variants were checked or adjusted to ensure that they matched with the reference transcript NM_001376.5 for *DYNC1H1*.

### Analysis and Interpretation of *DYNC1H1* Variants

Sanger sequencing was performed to confirm the mutations and co-segregations within family members. Various *in silico* algorithms were applied to interpret the pathogenicity of detected variants, including MutationTaster (http://www.mutationtaster.org/), PolyPhen-2 (http://genetics.bwh.harvard.edu/pph2/), SIFT (http://provean.jcvi.org/genome_submit_2.php?species=human), and PROVEAN (http://provean.jcvi.org/genome_submit_2.php?species=human). The pathogenicity of gene variations was classified according to the classification standard of the American College of Medical Genomics (ACMG) (10). Protein sequences across species were aligned using the ClustalW algorithm *via* MEGA11 software.

### Collection of Previous Publications

Online databases, including PubMed, Web of Science, CNKI, and Wanfang Data, were searched, applying the following search terms from January 1980 to April 2021: (mutation OR variant) AND [DYNC1H1 AND (“Charcot-Marie-Tooth Disease” OR “CMT” OR “hereditary motor and sensory neuropathy” OR “HMSN” OR “spinal muscular atrophy” OR “SMA”)]. Human Gene Mutation Database (HGMD) (http://www.hgmd.cf.ac.uk/), Online Mendelian Inheritance in Man (OMIM) (https://www.omim.org/), and Ensembl (http://uswest.ensembl.org/index.html) were also searched with the search term *DYNC1H1*. All resulting publications were carefully screened. We excluded publications that (1) were reviews, (2) were laboratory studies, (3) were not related to neuromuscular diseases, and (4) did not involve any *DYNC1H1* variants. Among the included publications, families or a single patient with no detailed description of clinical phenotypes were also excluded from this study to improve the quality of analysis. Clinical and genetic data were collected from each reported patient. All the variants were checked or adjusted to ensure that they matched with the reference transcript NM_001376.5 for *DYNC1H1*. All the processes were performed independently by two authors (J.L. and S.D.), and any discrepancy in the assessment were resolved by consensus.

### Statistics

The distributions of continuous variables were tested for normality using the Kolmogorov–Smirnov test, and the difference between the two groups was compared using Student's *t*-test or the Mann–Whitney test, wherever appropriate. The chi-squared test or the Fisher exact test was used to compare the categorical variables. Moreover, Bonferroni adjustment was also applied in multiple comparisons. All analyses were two-tailed, and a *p-*value <0.05 was considered statistically significant. All the statistical data were analyzed using SPSS 20.0 (SPSS Inc., Chicago, IL, USA).

## Results

### Clinical and Genetic Analysis of Two SMALED1 Cases

**Figure 1:**
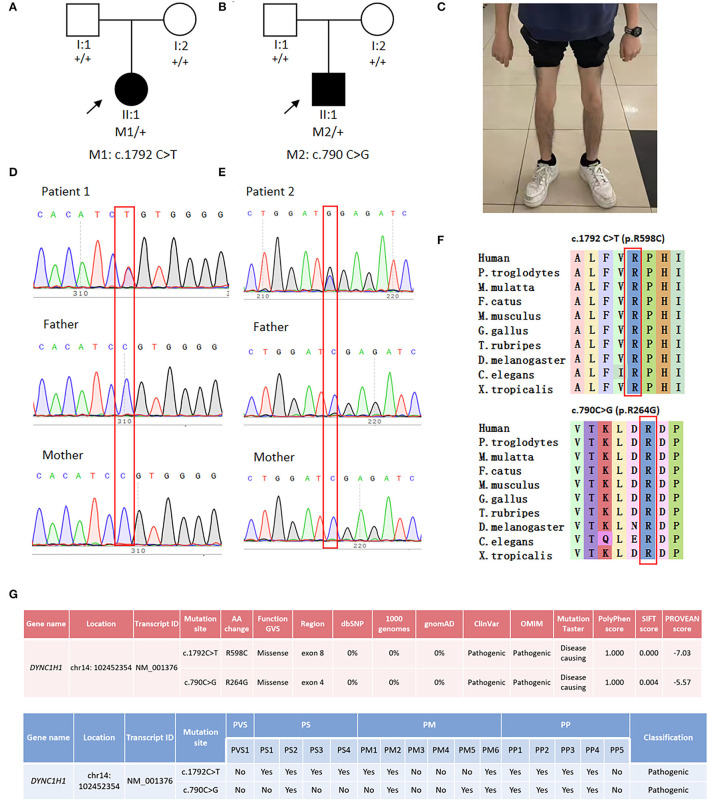
Genetic analysis and clinical phenotypes of two SMALED1 pedigrees. **(A)** Family tree of patient 1. **(B)** Family tree of patient 2. **(C)** Patient 2 presented with obvious muscle wasting in both distal lower limbs. **(D)** Sanger sequencing of Patient 1 and her parents revealed a *de novo DYNC1H1* gene mutation c.1792C>T. **(E)** Sanger sequencing of patient 2 and his parents revealed a *de novo DYNC1H1* gene mutation c.790C>G; the affected member is marked in black, and the proband is indicated by arrows. **(F)** Affected amino acids R598 and R264 were conserved in human and various other vertebrates. **(G)** Pathogenicity prediction of the mutations by several *in silico* bioinformatic tools and according to ACMG guidelines.

Case 1 is a 14-year-old female patient who complained of difficulty standing upright from a squatting position for over 1 year. The patient learned to walk at 14 months of age and ran slower than her peers since childhood. On physical examination, the patient presented with bilateral *pes cavus* and muscle wasting of distal lower limbs. Muscle strength was normal, except for 4/5 weakness of bilateral distal upper limbs and lower limbs. Tendon reflexes were attenuated in all limbs, and Babinski's sign was negative. The sensory system was normal on examination. The patient also showed an unaffected intelligence level. Her parents had normal manifestations, and there was no related family history (Figure 1A).

Case 2 is a 15-year-old male patient who sought clinical help due to the noticeable thinning of lower limbs for 2 years. The patient could walk since 19 months of age and had been prone to fall since childhood. Physical examination showed mild lordosis and muscle wasting of both lower limbs (Figure 1C). Muscle strength was normal, except for 4/5 weakness of bilateral distal lower limbs. Tendon reflexes were attenuated. Babinski's sign was absent. The patient's sensory examination was normal with an unaffected intelligence level. His parents were both normal, and no related family history was declared (Figure 1B).

Laboratory examinations revealed normal (181 U/L) and elevated (294 U/L) levels of serum creatine kinase (CK) in the two patients, respectively. In case 1, electromyogram (EMG) detected no fibrillation potential or positive sharp wave in the right lingualis, the trapezius, the rectus abdominis, the iliopsoas, the vastus medialis, the medial head of the musculi gastrocnemius, the tibialis anterior, the first dorsal interossei, the flexor carpi radialis, or the musculus biceps brachii. However, widened motor unit potentials (MUPs) and decreased recruitment were found during slight and strong contractions in the aforementioned muscles, except for the right lingualis and the right rectus abdominis. The nerve conduction studies (NCSs) revealed normal nerve conduction velocity (NCV), compound muscle action potential (CMAP) amplitude, and sensory nerve action potential (SNAP) amplitude in the right median, peroneal, ulnar, and tibial nerves. In case 2, EMG detected a positive sharp wave in the right tibialis anterior muscle, as well as giant potentials during slight contraction in the right sternocleidomastoid, the trapezius, the rectus abdominis, the musculus biceps brachii, the flexor carpi radialis, the first dorsal interossei, the medial head of the musculi gastrocnemius, the tibialis anterior, and the bilateral vastus medialis. In addition, decreased recruitment in the aforementioned muscles, except for the right sternocleidomastoid and the right first dorsal interossei, and increased polyphasic waves in the right musculi gastrocnemius were also detected. NCS was normal, except for the failure of SNAP induction in the left sural nerve. In both cases, the F wave latency showed normal results in the right median, peroneal, ulnar nerves and the bilateral tibial nerve. Both the M-latency and H-latency on the H reflex test of the bilateral tibial nerve were within normal range. The repetitive nerve stimulation (RNS) test was performed on the right abductor pollicis brevis, the tibialis anterior, and the trapezius with both low-frequency and high-frequency stimulations, which resulted in normal amplitude decay and area decay. In the second patient, magnetic resonance imaging (MRI) of the femur showed a partial fat signal, suggesting muscle atrophy of the right thigh muscle group and a small amount of effusion in the right hip joint cavity. Biopsy of the left sural nerve revealed that the pathological changes were consistent with pathological characteristics of chronic axonal peripheral neuropathy.

WES discovered *DYNC1H1* c.1792C>T (p.R598C) and *DYNC1H1* c.790C>G (p.R264G) heterozygous mutations in the two patients, respectively. The former mutation was reported in the East Asian population for the first time, and the latter was a novel mutation site. Sanger sequencing confirmed the variants in both probands and revealed no such mutations in their biological parents (Figures 1D,E), indicating these mutations *de novo*. The affected amino acids were conserved across vertebrates (Figure 1F). Various bioinformatics software packages (Mutation Taster, Polyphen2, SIFT, and PROVEAN) were applied to examine the pathogenicity of the two mutations. Both mutations were predicted as “pathogenic” according to ACMG guidelines (Figure 1G).

### *DYNC1H1* Mutations in Neuromuscular Diseases

Previous publications were searched to further explore the clinical and genetic spectrums of *DYNC1H1*-related neuromuscular diseases. A total of 22 original articles out of 49 publications met the inclusion criteria and were included in our study (Supplementary Figure 1). These articles identified 39 distinct variants in *DYNC1H1* related to neuromuscular diseases. Of the 105 patients with *DYNC1H1* mutations reported by these publications, SMALED1 (77, 73.3%) and CMT2O (16, 15.2%) accounted for the most part. Among the patients with SMALED1, two were initially diagnosed with polio and chronic inflammatory demyelinating polyradiculoneuropathy (CIDP). Other types of neuromuscular diseases, such as hereditary spastic paraplegia (HSP), were also reported to be caused by *DYNC1H1* variants. Of all the 58 families reported, most were European families (34/58, 58.6%), followed by East Asia (9/58, 15.5%), North America (8/58, 13.8%), Australia (5/58, 8.6%), and the Middle East (2/58, 3.4%).

### Mutational Spectrum of *DYNC1H1* Associated With Neuromuscular Diseases

**Figure 2:**
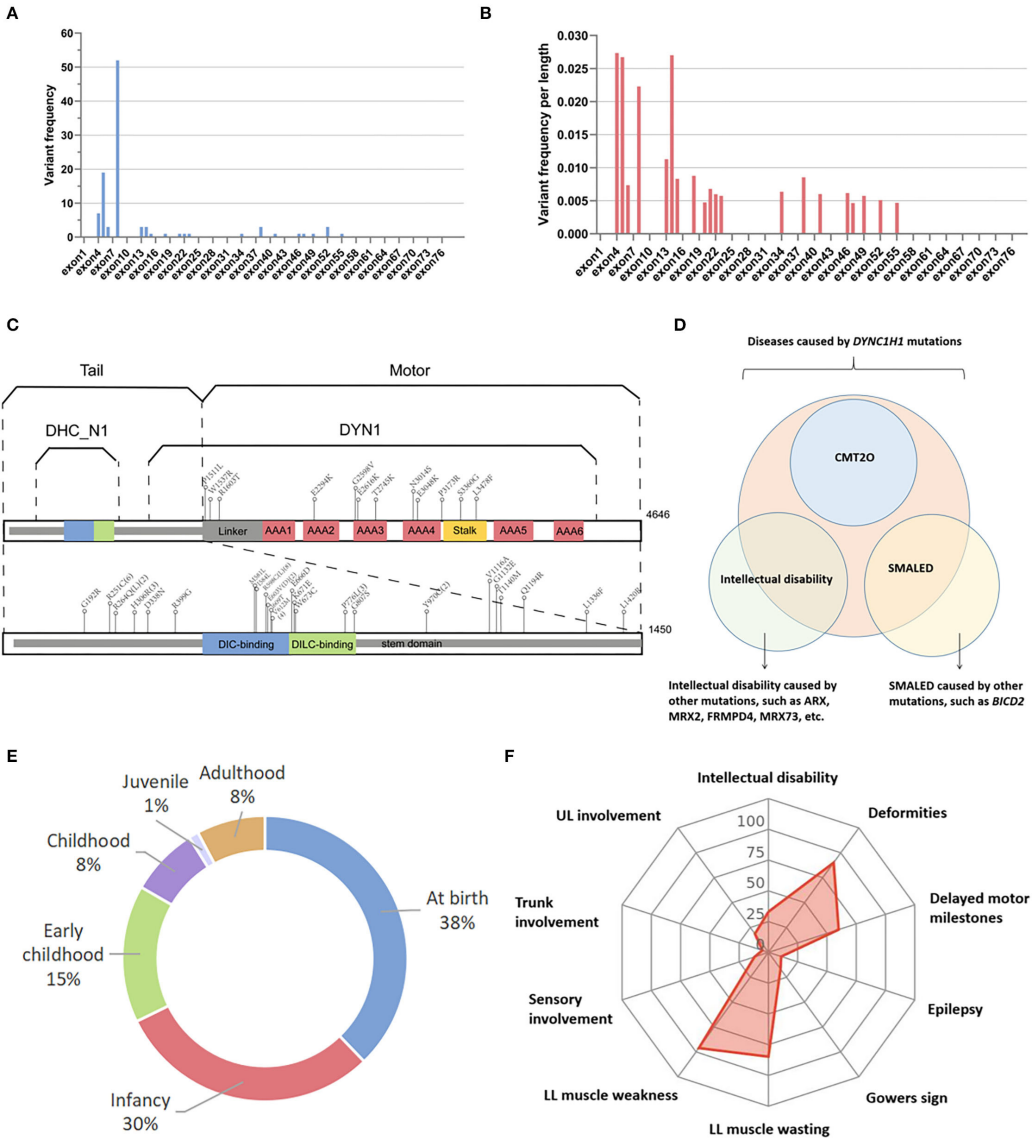
Genetic and phenotypic spectrum of neuromuscular diseases caused by *DYNC1H1* mutations. **(A)** *DYNC1H1* variants on different exons not adjusting for exon length. **(B)** *DYNC1H1* variants on different exons adjusting for exon length by calculating variant amounts per length. **(C)** Structural model of DYNC1H1 protein and relevant mutation sites in previous publications associated with neuromuscular diseases; the numbers in brackets indicate the recurrent times of mutations in different pedigrees; **(D)** Relationship between SMALED1, CMT2O, and *DYNC1H1* mutations. **(E)** Distribution of disease onset age of patients with neuromuscular diseases caused by *DYNC1H1* mutations; **(F)** Radar chart shows the percentage of some common features of *DYNC1H1-*related neuromuscular diseases. AAA, ATPase family associated with various cellular activities; ARX, aristaless-related homeobox; BICD2, BICD cargo adaptor 2; CMT2O, Charcot–Marie tooth type 2O; DIC, dynein intermediate chain; DILC, dynein intermediate light chain; DLL, distal lower limbs; FRMPD4, FERM, and PDZ domain containing 4; LL, lower limbs; PLL, proximal lower limbs; SMALED, spinal muscular atrophy with lower extremity predominance; UL, upper limbs.

All variants were missense mutations, except for one splicing variant (c.12685-3C>T); two patients from a Chinese family had complex mutations in *DYNC1H1* (c.2419G>A and c.12685-3C>T). To analyze the exon distribution of the missense variants, the average frequency of variants per length was calculated to balance the length of exon variation. Analysis of *DYNC1H1* variants revealed that exon 8 carried the most amount of *DYNC1H1* variants (Figure 2A). However, after adjusting for exon length, we found that exons 4, 14, 5, and 8 were the top four hot regions related to neuromuscular diseases, with variants per length estimated to be 0.0273, 0.0270, 0.0267, and 0.0223, respectively (Figure 2B). Several recurrent *DYNC1H1* variants were reported, with c.1792C>T (p.R598C) being the most common mutation (7/58 families with 10 patients, 12.1%). Others were c.751C>T (6/58, 10.3%), c.1953G>A (4/58 6.9%), c.2327C>T (3/58, 5.2%), c.917A>G (3/58, 5.2%), and c.3170A>G (2/58, 3.4%). Interestingly, although c.1792C>T (p.R598C) was suggested to be the most common mutation site related to neuromuscular diseases worldwide, it has not been reported in the East Asian population in previous publications. Instead, c.751C>T (3/9 families, 33.3%) and c.2327C>T (2/9 families, 22.2%) were more commonly reported in this area.

Human DYNC1H1 protein is composed of 4646 amino acids (aa). It can be roughly divided into the tail region (0-1450 aa) and the motor region (1450-4646 aa) according to its structural and functional characteristics (11). Despite that the motor region was longer, 27 of the 39 variants (69.2%) were distributed in the tail region, and only 12 of the 39 (30.8%) were located in the motor region, which was in correspondence with previous studies that mutations in the tail region were more commonly related to motor-related disorders (4, 12). More precisely, the DYNC1H1 protein is divided into several domains overlapping each other (Figure 2C). Cases involved in our study revealed that the stem domain (53-1867 aa) contained the most amount of DYNC1H1 mutations (30/39, 76.9%), among which 18 of the 39 (46.2%) were located in overlapping regions of the stem with other domains, including DHC_N1 (242-832 aa), dynein intermediate chain (DIC)-binding domain (448-703 aa), and dynein intermediate light chain (DILC)-binding domain (651-802 aa). Mutations in other domains were relatively less frequent, including three ATPase families associated with various cellular activity (AAA) domains (2180-3168 aa, 7/39, 17.9%) and stalk (3189-3500 aa, 2/39, 5.1%) (Figure 2C). This result may be due to the fact that the stem is the longest domain of DYNC1H1 located more closely to the N terminal and overlaps with many other regions. The detailed information on the *DYNC1H1* genetic spectrum of patients with neuromuscular diseases is listed in Supplementary Table 1.

Apart from neuromuscular diseases such as SMALED1 and CMT2O, *DYNC1H1* gene mutation is also related to intellectual disability characterized by early-onset seizures, mild dysmorphic features, and cortical malformations (13–15). CMT2O is uniquely linked with *DYNC1H1* gene mutations, while SMALED has also been reported to be associated with *BICD cargo adaptor 2 (BICD2)* mutations (8, 16). BICD2 protein is important in dynein complex binding, and animal models indicated that *BicD2* knockdown mouse embryos showed significant inhibition of neuronal migration (8, 17). The relationship between SMALED, CMT2O, and *DYNC1H1* mutations is illustrated in Figure 2D.

### Clinical Spectrum of Neuromuscular Diseases Caused by *DYNC1H1* Mutations

A total of 105 patients with neuromuscular diseases were reported carrying *DYNC1H1* mutations. Of 66 patients with exact ages provided, the average age was 21.8±19.7 (range, 1.0–82.0) years. The onset age of the patients with *DYNC1H1* mutations was relatively young, with more than two-thirds of the patients showing disease onset at birth or in infancy (<1 year old) (Figure 2E). The detailed clinical information of patients with neuromuscular diseases caused by *DYNC1H1* mutations is listed in Supplementary Table 2. Muscle weakness of lower limbs was the most common clinical phenotype of these patients, among which nearly two-thirds presented with proximal dominant weakness of lower limbs. In comparison, more patients showed distal dominant atrophy than proximal dominant atrophy. This may be due to the larger volume of proximal muscles in the lower limbs, which can hide the relatively small degree of proximal muscle wasting. A small part of the patients also had upper limb involvement. Delayed motor milestones were usually the earliest clinical manifestation of patients with SMALED1 or CMT2O and were reported in more than half of the patients. However, most patients remained ambulant, and Gowers sign was not common, suggesting the relatively mild degree of lower limb impairment and the rare involvement of trunk muscle in these groups of diseases. The sensory system was usually exempted from the diseases, and the involvement of the central nervous system (CNS) was also relatively less common, with around one-third of patients having intellectual disability and around one-tenth of them experiencing seizure episodes. Most patients showed deformities predominantly involving the musculoskeletal system, of which *pes cavus* and joint contracture or deformities were the most common. Other deformities, including *pes planus*, hand deformities, spine deformities, scapular winging, exotropia, and aortic root dilation were also observed in a number of patients. The percentage of some clinical phenotypes is shown in Figure 2F.

Decreased or absent tendon reflex was the most notable clinical phenotype on physical examination. The serum CK level and NCV test showed abnormal results only in a small percentage of patients. Abnormal brain MRI was more common and was detected in about 60% of patients.

### Genotype–Phenotype Correlations of *DYNC1H1* Variants

To further explore the phenotypic and genetic spectrum of *DYNC1H1* mutations, we then analyzed the genotype–phenotype correlations between *DYNC1H1* variants and neuromuscular diseases. First, we analyzed whether there was any correlation between the types of *DYNC1H1* mutations and the kinds of neuromuscular diseases. However, no significant correlation between diagnosis and mutation site was found (Table 1).

**Table 1:** Comparison of mutation site distribution between SMALED1 and CMT2O families.

**Diagnosis**	**Total families**	**Mutation sites**	**Fisher exact test**	
		**DHC_N1 domain (%)**	**DYN1 domain (%)**	***P*** **-value**
SMALED1	43	35 (81.4)	8 (18.6)	0.488
CMT2O	3	2 (66.7)	1 (33.3)	

*CMT2O, Charcot–Marie-Tooth type 2O; SMALED1, spinal muscular atrophy with lower extremity predominance 1*.

**Figure 3:**
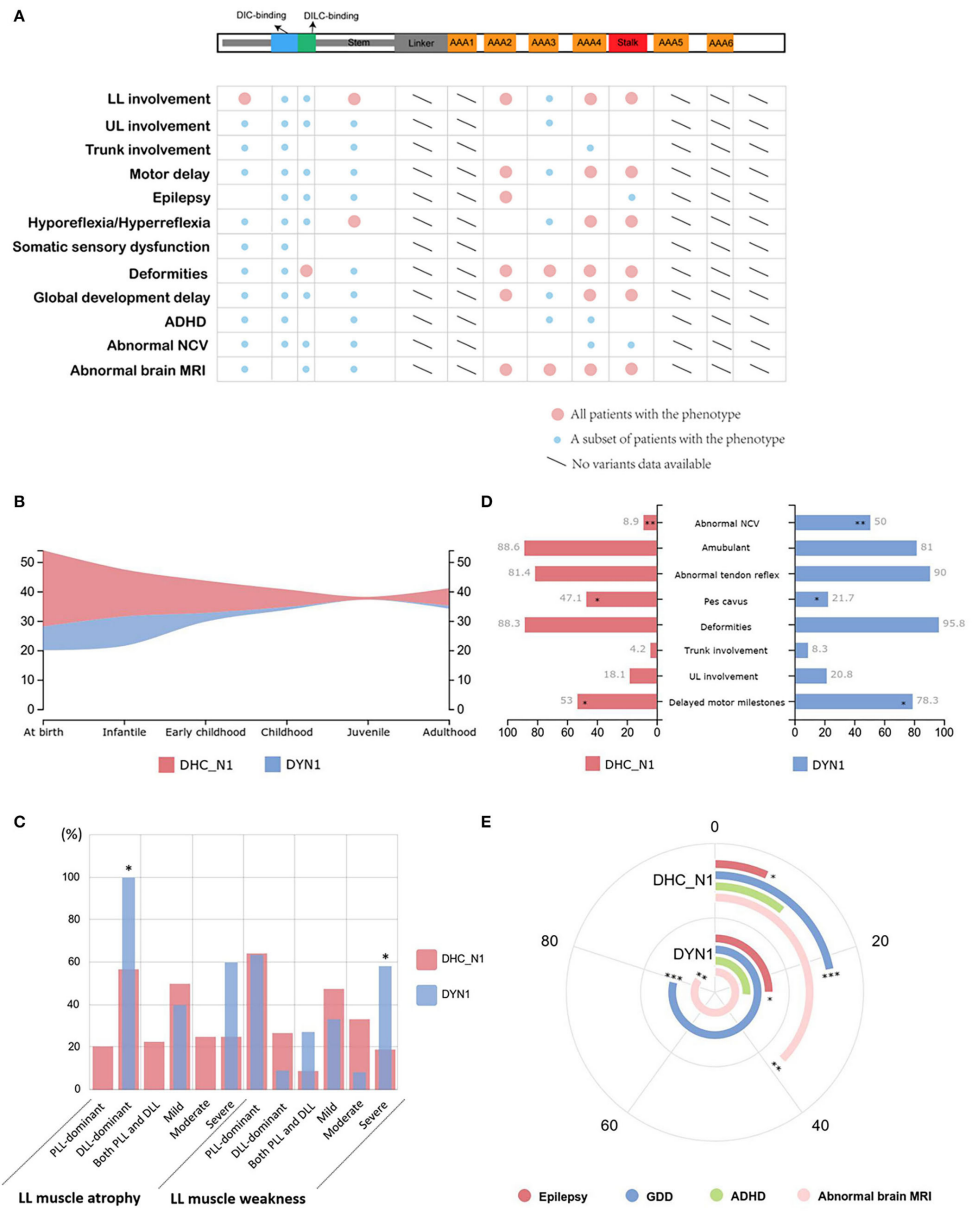
Genotype–phenotype correlation of *DYNC1H1* mutations. **(A)** Correlations between mutations in different domains and clinical manifestations of neuromuscular diseases. The large red circle indicates that all patients carrying DYNC1H1 mutations in a specific domain have the relevant phenotype labeled on the left side. The small blue circle refers to a small subset of patients who have the phenotype. **(B)** Comparison of the distribution pattern of onset age between the DHC_N1 group and the DYN1 group. **(C)** Comparison of the percentage of muscular manifestations between the two groups. *P < 0.05. **(D)** Comparison of the percentage of other common manifestations between the two groups. *P < 0.05, **P < 0.01. **(E)** Comparison of the percentage of CNS manifestations between the two groups. The angle corresponding to each arch represents the relative percentage of each manifestation. *P < 0.05, **P < 0.01, ***P < 0.001. AAA, ATPase family associated with various cellular activities; ADHD, attention deficit and hyperactivity disorder; DIC, dynein intermediate chain; DILC, dynein intermediate light chain; DLL, distal lower limb; GDD, global developmental delay; LL, lower limb; MRI, magnetic resonance imaging; NCV, nerve conduction velocity; PLL, proximal lower limb; UL, upper limb.

Next, we analyzed the correlation between clinical phenotypes of neuromuscular diseases and different mutation sites of DYNC1H1 protein. As illustrated in Figure 3A, all patients carrying DYNC1H1 mutations in the motor domain (including AAA2, AAA3, AAA4, and stalk) presented with deformities and abnormal brain MRI. In addition, most DYNC1H1 mutations in the motor domain are correlated with abnormal brain functions, such as epilepsy and global development delay. These phenotypes were relatively less common in patients with mutations in the DYNC1H1 tail domain. To further quantify the genotype–phenotype correlations, we divided the DYNC1H1 protein into three parts: the N-terminal region named DHC_N1 (242–832 aa), the C-terminal region named Dynein_C (4333–4633 aa), and the longest DYN1 region (1046-4329 aa) in between. Of all the DYNC1H1 mutations leading to neuromuscular diseases in the current study, none were located in Dynein_C. Therefore, the mutations can be further divided into two comparable groups according to their locations: the DHC_N1 group (p.G192R also included) and the DYN1 group (p.Y970C also included). The disease onset age distribution was relatively similar between the two groups (Figure 3B). Atrophy and weakness of lower limbs were both common in the two groups, and both groups had more cases of distal-dominant atrophy and proximal-dominant weakness. However, the severity of these symptoms between the two groups showed some differences, with more severe lower limb weakness lying in the DYN1 group than in the DHC_N1 group (7/12, 58.3% vs. 4/21, 19.0%; *P* < 0.05) (Figure 3C). Delayed motor milestones were more commonly observed in the DYN1 group than in the DHC_N1 group (18/23, 78.3% vs. 35/66, 53.0%, *P* = 0.034) (Figure 3D). In addition, the DYN1 group also had a higher percentage of upper limb involvement, trunk involvement, deformities, and abnormal tendon reflex, as well as a lower percentage of ambulant patients, although the results were not statistically significant. However, *pes cavus* was significantly more common in the DHC_N1 group (32/68, 47.1% vs. 5/23, 21.7%, *P* = 0.033) (Figure 3D). Abnormal NCV results were also significantly more common in the DYN1 group than in the DHC_N1 group (6/12, 50% vs. 4/45, 8.9%, *P* = 0.004) (Figure 3D). These results indicated that mutations in the DYN1 region might present a more severe phenotype and more complicated clinical manifestations than the DHC_N1 mutations.

Mutations in the DYN1 region were more related to CNS impairment (Figure 3E). Higher number of epilepsy cases were observed in the DYN1 group (6/24, 25.0%) than in the DHC_N1 group (5/75, 6.7%; *P* = 0.034). The incidence of global developmental delay was significantly higher in the DYN1 group (15/19, 78.9% vs. 16/73, 21.9%, *P* < 0.001), and attention deficit and hyperactivity disorder (ADHD) was also more common in this group, although the difference was not significant. In accordance with this, abnormalities of the brain were more commonly found on MRI in the DYN1 group (12/14, 85.7%) than in the DHC_N1 group (6/16, 37.5%; *P* = 0.007).

## Discussion

*DYNC1H1* encodes heavy chain 1 of the dynein complex and is involved in the control of microtubule binding, as well as the recruitment of other dynein components (15, 18). In this study, we reported two SMALED1 cases with heterozygous *DYNC1H1* mutations c.1792C>T (p.R598C) and c.790C>G (p.R264G). The diagnosis of SMALED1 was supported by neuroelectrophysiological examinations, which revealed chronic denervation and reinnervation manifestations with the relatively preserved function of nerve conduction. The relative lack in active denervation potential was in accordance with the chronic course of these patients. Through further genotype–phenotype analysis, we found that patients with mutations in the DYN1 region may have a more severe phenotype, more complicated manifestations, and more CNS involvement than those who had mutations in the DHC_N1 region.

Covering a length of 19940 base pairs, *DYNC1H1* is a large gene with 78 exons. Although our study revealed that *DYNC1H1* variants were widely dispersed across the whole gene, it was notable that exons 4, 14, 5, and 8 were the mutational hot regions of *DYNC1H1* after adjusting for exon length in this study. A previous study including 14 families with *DYNC1H1* mutations found that 13 of the 14 families had mutations in the tail domain of *DYNC1H1*. Nevertheless, a cluster of mutations occurred in exon 8 within the tail domain itself (19). This conclusion might be due to the fact that exon 8 is the longest encoding exon of the *DYNC1H1* gene, as our study also showed that exon 8 contained the most mutations while not adjusting for exon length. Despite the discrepancy between our study and previous research, all the mutational hot regions (including exons 4, 5, 8, and 14) were in the DHC_N1 region of the tail domain. The tail domain lies in the N terminal of the dynein heavy chain and contains binding positions for dynein intermediate chain (DIC) and dynein intermediate light chain (DILC), the latter of which further provides a separate binding site for dynein light chain (DLC). The DIC, DILC, and DLC together form the cargo-binding site of the dynein complex (20). Therefore, mutations in the tail domain affect the binding capacity of the dynein heavy chain with other components, which can lead to neurological impairment possibly *via* disrupting the retrograde transport of activated neurotrophin receptors (21, 22) and nerve injury signals (23, 24) or by an interruption in the clearance of misfolded proteins by autophagy (25). Of the mutational hot regions in this study, c.1792C>T was the most frequent point mutation, which was predicted to replace an arginine with a cysteine at the DIC-interacting site. However, no c.1792C>T mutation has been found in the East Asian population in previous publications. On the one hand, this discrepancy may reflect differences at the genetic level among different races; on the other hand, this result may be due to the relatively limited amount of cases in this area. Considering the large population basis in East Asia, more different kinds of *DYNC1H1* mutations may be gradually detected, as we reported a teenage patient with SMALED1 caused by *DYNC1H1* c.1792C>T heterozygous mutation in this study. Notably, c.751C>T (p.R251C) and c.2327C>T (p.P776L) were the most commonly reported mutations in East Asia. These two mutation sites lie in exon 4 and exon 8, which provides further support for the conclusion that these exons are hot regions of *DYNC1H1* mutations. Recent years have witnessed significant improvement in gene sequencing techniques, and the potential of gene therapy has been trialed in treating various incurable hereditary diseases. Considering the highly dispersed distribution of *DYNC1H1* mutations, precise base editing may not be practical for correcting a *DYNC1H1* mutation in most patients; however, specific editing of the hotspots in the mutational hot regions has a promising future (26).

To our knowledge, few studies have been devoted to unraveling the genotype–phenotype relationship between neuromuscular disorders and *DYNC1H1* variants. In the current study, mutations in the DYN1 region of DYNC1H1 protein were found to be correlated with more severe clinical phenotypes, more complicated manifestations, and more common CNS impairment than variants in the DHC_N1 region. This is in accordance with earlier findings that mutations in the tail domain (close to the N terminal) usually lead to pure motor neuron deficits hardly with any signs of brain abnormalities (4, 12), while mutations in the motor domain (close to the C terminal) often cause the malformation of cortical development (MCD) and epilepsy (15). The reasons underlying this phenotypic diversity have not been clearly elucidated yet. One study based on the yeast model system suggested that the degree of genetic dysfunction may be a potent determinant of phenotypic type. In this study, Matthew et al. revealed that motor system deficits seem to be susceptible to a small degree of dynein dysfunction, while brain function abnormalities appear to be associated with larger degrees of dysfunction (3). In precision, the coefficient of dynein dysfunction (CDD) for motor neuron diseases is from 5 to 18, smaller than the CDD for malformation of cortical development, which is ≥ 19 (3). Given that, in our study, different severity of clinical manifestations is correlated with different DYNC1H1 protein regions that mutations are located on, it is deduced that different mutational regions may lead to divergence in the degrees of a genetic dysfunction, which finally causes variability in the severity of dynein protein abnormalities. Further functional studies of the genetic characteristics at different sites of dynein protein will help us to explore the reason for the phenotypic heterogeneity.

The conclusions of this study should not be assessed without the consideration of several limitations. First, the study did not present a complete mutation spectrum since patients without any detailed description of clinical features were excluded from this study. Second, the reporting bias of included articles should not be ignored because cases with recurrent variants and classical phenotypes were less likely to be reported. Third, all these comparisons are based on the literature and online databases with a relatively limited amount of cases, and the genotype–phenotype correlations should be validated in the larger patient population in future. Despite the limitations, our study provided a comprehensive summary of the *DYNC1H1* mutation spectrum by selecting relevant neuromuscular diseases, especially SMALED1 and CMT2O, and unraveled the hidden correlation between genotypes and phenotypes.

## Conclusion

In this study, we reported novel *DYNC1H1* c.790C>G (p.R264G) heterozygous mutations that caused SMALED1. We provided a detailed description of the clinical and genetic spectrum of neuromuscular diseases caused by *DYNC1H1* mutations by reviewing previous publications. Our findings also suggest that mutations in the DYN1 region of DYNC1H1 may be correlated with more severe clinical phenotypes, more complicated manifestations, and more common CNS involvement, which may shed light on unraveling the genetic mechanism underlying clinical heterogeneity of “dyneinopathy.”

## Data Availability Statement

The datasets presented in this study can be found in online repositories. The name of the repository and accession number(s) can be found below: National Center for Biotechnology Information (NCBI) GenBank, https://www.ncbi.nlm.nih.gov/genbank/, ON548480–ON548485.

## Ethics Statement

The studies involving human participants were reviewed and approved by the Ethics Committee of Huashan Hospital, Fudan University. Written informed consent was obtained from the individual(s), and minor(s)' legal guardian/next of kin, for the publication of any potentially identifiable images or data included in this article.

## Author Contributions

J-TL and S-QD completed the collection and assessment of previous publications independently and wrote the manuscript. X-JC revised the manuscript. All authors designed the study, collected the clinical and genetic data of the two patients, read, and approved the manuscript.

## Funding

This study was supported by the Open Project of State Key Laboratory of Cell Biology (No. SKLCB2018KF004), the 2020 Central Transfer Payment Medical Siege Institutions Capacity Building Project (National and Provincial Multi-scientific Cooperation Diagnosis and Treatment of Major Diseases Capacity Building Project), and Shanghai Fudan University Education Development Foundation, and the State Key Laboratory of Genetic Engineering, Human Phenome Institute, Zhangjiang Fudan International Innovation Center, Fudan University.

## Conflict of Interest

The authors declare that the research was conducted in the absence of any commercial or financial relationships that could be construed as a potential conflict of interest.

## Publisher's Note

All claims expressed in this article are solely those of the authors and do not necessarily represent those of their affiliated organizations, or those of the publisher, the editors and the reviewers. Any product that may be evaluated in this article, or claim that may be made by its manufacturer, is not guaranteed or endorsed by the publisher.

## Supplementary Material

Supplementary Figure 1 Flow chart of publication screening process. A total of 22 previous publications out of 49 original articles were finally included according to the inclusion and exclusion criteria.

Supplementary Table 1 DYNC1H1 variants associated with neuromuscular diseases in previous publications.

Supplementary Table 2 Demographics and clinical characteristics of 105 patients with DYNC1H1 variants.
