# Supplementary figures and images for: Expanding the Phenotypic and Genetic Spectrum of Neuromuscular Diseases Caused by DYNC1H1 Mutations
Source: Front Neurol. 2022 Jul 11;13:943324. doi: 10.3389/fneur.2022.943324 (PMC9309508; doi:10.3389/fneur.2022.943324)

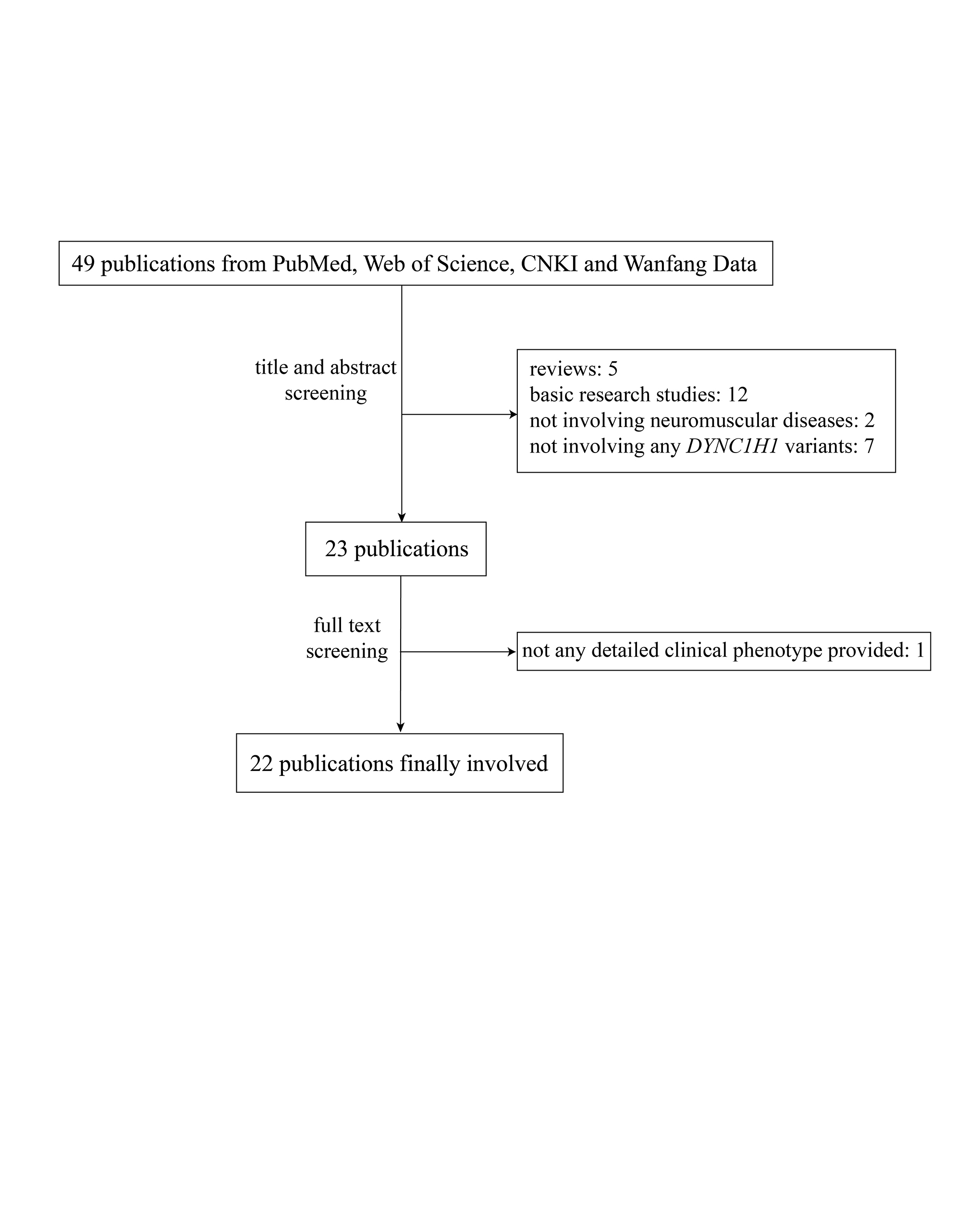

Supplement: Supplementary Figure 1 — Flow chart of publication screening process. A total of 22 previous publications out of 49 original articles were finally included according to the inclusion and exclusion criteria. [file Image_1.JPEG]
