# Supplementary material for: Expanding the Phenotypic and Genetic Spectrum of Neuromuscular Diseases Caused by DYNC1H1 Mutations
Source: Front Neurol. 2022 Jul 11;13:943324. doi: 10.3389/fneur.2022.943324 (PMC9309508; doi:10.3389/fneur.2022.943324)
Supplement: Supplementary Table 1 — DYNC1H1 variants associated with neuromuscular diseases in previous publications. [file Table_1.DOCX]

**Supplementary Table 1. *DYNC1H1* variants associated with neuromuscular diseases in previous publications**

| **Reference** | **family ID** | **patient ID** | **Mutation site** | **Mutation amounts** | **Exon** | **Domain** |
| --- | --- | --- | --- | --- | --- | --- |
| Beecroft et al, 2017 | 1 (part*) | 1 | c.1792C>T,p.R598C | 1 | 8 | DHC_N1, stem, DIC-interacting |
| Beecroft et al, 2017 |  | 2 | c.1792C>T,p.R598C | 1 | 8 | DHC_N1, stem, DIC-interacting |
| Beecroft et al, 2017 | 2 | 3 | c.2327C>T,p.P776L | 1 | 8 | DHC_N1, stem, DLIC-interacting |
| Beecroft et al, 2017 | 3 | 4 | c.1792C>T,p.R598C | 1 | 8 | DHC_N1, stem, DIC-interacting |
| Beecroft et al, 2017 | 4 | 5 | c.1792C>T,p.R598C | 1 | 8 | DHC_N1, stem, DIC-interacting |
| Viollet et al, 2020 | 5 (part) | 6 | c.1826T>C,p.I609T | 1 | 8 | DHC_N1, stem, DIC-interacting |
| Viollet et al, 2020 |  | 7 | c.1826T>C,p.I609T | 1 | 8 | DHC_N1, stem, DIC-interacting |
| Viollet et al, 2020 |  | 8 | c.1826T>C,p.I609T | 1 | 8 | DHC_N1, stem, DIC-interacting |
| Viollet et al, 2020 |  | 9 | c.1826T>C,p.I609T | 1 | 8 | DHC_N1, stem, DIC-interacting |
| Viollet et al, 2020 |  | 10 | c.1826T>C,p.I609T | 1 | 8 | DHC_N1, stem, DIC-interacting |
| Tsurusaki et al, 2012 | 6 | 11 | c.917A>G, p.H306R | 1 | 5 | DHC_N1, stem |
| Tsurusaki et al, 2012 |  | 12 | c.917A>G, p.H306R | 1 | 5 | DHC_N1, stem |
| Tsurusaki et al, 2012 |  | 13 | c.917A>G, p.H306R | 1 | 5 | DHC_N1, stem |
| Harms et al, 2012 | 7 (part) | 14 | c.2011A>G,p.K671E | 1 | 8 | DHC_N1, stem, DIC-interacting, DLIC-interacting |
| Harms et al, 2012 | 8 | 15 | c.3170A>G,p.Y970C | 1 | 12 | stem |
| Niu et al, 2015 | 9 | 16 | c.2419G>A,p.G807S c.12685-3C>T | 2 | 8 | stem |
| Niu et al, 2015 |  | 17 | c.2419G>A,p.G807S c.12685-3C>T | 2 | 8 | stem |
| Niu et al, 2015 |  | 18 | c.2419G>A,p.G807S | 1 | 8 | stem |
| Scoto et al, 2015 | 10 | 19 | p.R399G | 1 | 6 | DHC_N1, stem |
| Scoto et al, 2015 |  | 20 | p.R399G | 1 | 6 | DHC_N1, stem |
| Scoto et al, 2015 | 11 | 21 | p.R264Q | 1 | 4 | DHC_N1, stem |
| Scoto et al, 2015 | 12 | 22 | p.Y970C | 1 | 12 | stem |
| Scoto et al, 2015 |  | 23 | p.Y970C | 1 | 12 | stem |
| Scoto et al, 2015 | 13 | 24 | p.R598C | 1 | 8 | DHC_N1, stem, DIC-interacting |
| Scoto et al, 2015 |  | 25 | p.R598C | 1 | 8 | DHC_N1, stem, DIC-interacting |
| Scoto et al, 2015 | 14 | 26 | p.M581L | 1 | 8 | DHC_N1, stem, DIC-interacting |
| Scoto et al, 2015 | 15 | 27 | p.W673C | 1 | 8 | DHC_N1, stem, DIC-interacting, DLIC-interacting |
| Scoto et al, 2015 | 16 (part) | 28 | p.E603V | 1 | 8 | DHC_N1, stem, DIC-interacting |
| Scoto et al, 2015 | 17 | 29 | p.R1603T | 1 | 22 | stem |
| Scoto et al, 2015 | 18 | 30 | p.D338N | 1 | 6 | DHC_N1, stem |
| Scoto et al, 2015 | 19 | 31 | p.V612M | 1 | 8 | DHC_N1, stem, DIC-interacting |
| Scoto et al, 2015 |  | 32 | p.V612M | 1 | 8 | DHC_N1, stem, DIC-interacting |
| Scoto et al, 2015 |  | 33 | p.V612M | 1 | 8 | DHC_N1, stem, DIC-interacting |
| Scoto et al, 2015 |  | 34 | p.V612M | 1 | 8 | DHC_N1, stem, DIC-interacting |
| Scoto et al, 2015 |  | 35 | p.V612M | 1 | 8 | DHC_N1, stem, DIC-interacting |
| Scoto et al, 2015 | 20 (part) | 36 | p.I584L | 1 | 8 | DHC_N1, stem, DIC-interacting |
| Scoto et al, 2015 |  | 37 | p.I584L | 1 | 8 | DHC_N1, stem, DIC-interacting |
| Scoto et al, 2015 | 21 | 38 | p.V612M | 1 | 8 | DHC_N1, stem, DIC-interacting |
| Scoto et al, 2015 | 22 | 39 | p.V612M | 1 | 8 | DHC_N1, stem, DIC-interacting |
| Scoto et al, 2015 |  | 40 | p.V612M | 1 | 8 | DHC_N1, stem, DIC-interacting |
| Scoto et al, 2015 | 23 | 41 | p.V612M | 1 | 8 | DHC_N1, stem, DIC-interacting |
| Scoto et al, 2015 |  | 42 | p.V612M | 1 | 8 | DHC_N1, stem, DIC-interacting |
| Scoto et al, 2015 | 24 | 43 | p.R598L | 1 | 8 | DHC_N1, stem, DIC-interacting |
| Scoto et al, 2015 |  | 44 | p.R598L | 1 | 8 | DHC_N1, stem, DIC-interacting |
| Scoto et al, 2015 | 25 | 45 | p.E2616K | 1 | 38 | AAA3 |
| Scoto et al, 2015 |  | 46 | p.E2616K | 1 | 38 | AAA3 |
| Punetha et al, 2015 | 26 | 47 | c.1792C>T,p.R598C | 1 | 8 | DHC_N1, stem, DIC-interacting |
| Chen et al, 2017 | 27 | 48 | c.3395G>A,p.G1132E | 1 | 13 | stem |
| Chan et al, 2018 | 28 | 49 | c.751C>T, p.R251C | 1 | 3 | DHC_N1, stem |
| Chan et al, 2018 | 29 | 50 | c.751C>T, p.R251C | 1 | 3 | DHC_N1, stem |
| Chan et al, 2018 | 30 | 51 | c.751C>T, p.R251C | 1 | 3 | DHC_N1, stem |
| Chan et al, 2018 | 31 | 52 | c.751C>T, p.R251C | 1 | 3 | DHC_N1, stem |
| Das et al, 2018 | 32 | 53 | c.1809A>T,p.E603D | 1 | 8 | DHC_N1, stem, DIC-interacting |
| Das et al, 2018 |  | 54 | c.1809A>T,p.E603D | 1 | 8 | DHC_N1, stem, DIC-interacting |
| Das et al, 2018 |  | 55 | c.1809A>T,p.E603D | 1 | 8 | DHC_N1, stem, DIC-interacting |
| Das et al, 2018 |  | 56 | c.1809A>T,p.E603D | 1 | 8 | DHC_N1, stem, DIC-interacting |
| Das et al, 2018 |  | 57 | c.1809A>T,p.E603D | 1 | 8 | DHC_N1, stem, DIC-interacting |
| Das et al, 2018 |  | 58 | c.1809A>T,p.E603D | 1 | 8 | DHC_N1, stem, DIC-interacting |
| Weedon et al, 2011 | 33 (part) | 59 | c.917A>G, p.H306R | 1 | 5 | DHC_N1, stem |
| Weedon et al, 2011 |  | 60 | c.917A>G, p.H306R | 1 | 5 | DHC_N1, stem |
| Weedon et al, 2011 |  | 61 | c.917A>G, p.H306R | 1 | 5 | DHC_N1, stem |
| Weedon et al, 2011 |  | 62 | c.917A>G, p.H306R | 1 | 5 | DHC_N1, stem |
| Weedon et al, 2011 |  | 63 | c.917A>G, p.H306R | 1 | 5 | DHC_N1, stem |
| Weedon et al, 2011 |  | 64 | c.917A>G, p.H306R | 1 | 5 | DHC_N1, stem |
| Weedon et al, 2011 |  | 65 | c.917A>G, p.H306R | 1 | 5 | DHC_N1, stem |
| Weedon et al, 2011 |  | 66 | c.917A>G, p.H306R | 1 | 5 | DHC_N1, stem |
| Weedon et al, 2011 |  | 67 | c.917A>G, p.H306R | 1 | 5 | DHC_N1, stem |
| Weedon et al, 2011 |  | 68 | c.917A>G, p.H306R | 1 | 5 | DHC_N1, stem |
| Weedon et al, 2011 |  | 69 | c.917A>G, p.H306R | 1 | 5 | DHC_N1, stem |
| Weedon et al, 2011 |  | 70 | c.917A>G, p.H306R | 1 | 5 | DHC_N1, stem |
| Weedon et al, 2011 |  | 71 | c.917A>G, p.H306R | 1 | 5 | DHC_N1, stem |
| Argente-Escrig et al, 2020 | 34 | 72 | c.751C>T, p.R251C | 1 | 3 | DHC_N1, stem |
| Argente-Escrig et al, 2020 | 35 | 73 | c.917A>G, p.H306R | 1 | 5 | DHC_N1, stem |
| Peeters et al, 2015 | 36 (part) | 74 | c.1792C>T,p.R598C | 1 | 8 | DHC_N1, stem, DIC-interacting |
| Peeters et al, 2015 |  | 75 | c.1792C>T,p.R598C | 1 | 8 | DHC_N1, stem, DIC-interacting |
| Peeters et al, 2015 | 37 | 76 | c.791G>T, p.R264L | 1 | 4 | DHC_N1, stem |
| Amabile et al, 2020 | 38 | 77 | c.3466T>C,p.V1116A | 1 | 13 | stem |
| Amabile et al, 2020 | 39 | 78 | c.4532C>T,p.P1511L | 1 | 20 | stem |
| Becker et al, 2020 | 40 | 79 | c.574G>A, p.G192R | 1 | 3 | stem |
| Becker et al, 2020 | 41 | 80 | c.10432C>T,p.L3478F | 1 | 54 | stalk |
| Becker et al, 2020 | 42 | 81 | c.4006C>T,p.L1336F | 1 | 17 | stem |
| Becker et al, 2020 | 43 | 82 | c.6880G>A,p.E2294K | 1 | 34 | AAA2 |
| Becker et al, 2020 | 44 | 83 | c.9041A>G p.N3014S | 1 | 46 | AAA4 |
| Becker et al, 2020 | 45 | 84 | c.4609T>C,p.W1537R | 1 | 21 | stem |
| Becker et al, 2020 | 46 | 85 | c.8234C>A,p.T2745K | 1 | 41 | AAA3 |
| Becker et al, 2020 | 47 | 86 | c.9518C>G,p.P3173R | 1 | 49 | between AAA4 and stalk |
| Becker et al, 2020 | 48 | 87 | c.7793G>T,p.G2598V | 1 | 38 | AAA3 |
| Becker et al, 2020 | 49 | 88 | c.1998A>T,p.E666D | 1 | 8 | DHC_N1, stem, DIC-interacting, DLIC-interacting |
| Fiorillo et al, 2014 | 50 | 89 | c.3581A>G,p.Q1194R | 1 | 15 | stem |
| Fiorillo et al, 2014 | 51 | 90 | c.9142G>A,p.E3048K | 1 | 47 | AAA4 |
| Strickland et al, 2015 | 52 | 91 | c.10078A>G,p.S3360G | 1 | 52 | stalk |
| Strickland et al, 2015 |  | 92 | c.10078A>G,p.S3360G | 1 | 52 | stalk |
| Strickland et al, 2015 |  | 93 | c.10078A>G,p.S3360G | 1 | 52 | stalk |
| Strickland et al, 2015 | 53 | 94 | c.1792C>T,p.R598C | 1 | 8 | DHC_N1, stem, DIC-interacting |
| Singh et al, 2015 | 54 | 95 | c.4259T>G,p.L1420R | 1 | 19 | stem |
| Ding et al, 2017 | 55 | 96 | c.2327C>T,p.P776L | 1 | 8 | DHC_N1, stem, DLIC-interacting |
| Ding et al, 2017 |  | 97 | c.2327C>T,p.P776L | 1 | 8 | DHC_N1, stem, DLIC-interacting |
| Wang et al, 2018 | 56 (part) | 98 | c.2327C>T,p.P776L | 1 | 8 | DHC_N1, stem, DLIC-interacting |
| Wang et al, 2018 |  | 99 | c.2327C>T,p.P776L | 1 | 8 | DHC_N1, stem, DLIC-interacting |
| Wang et al, 2018 |  | 100 | c.2327C>T,p.P776L | 1 | 8 | DHC_N1, stem, DLIC-interacting |
| Wang et al, 2018 |  | 101 | c.2327C>T,p.P776L | 1 | 8 | DHC_N1, stem, DLIC-interacting |
| Wang et al, 2018 |  | 102 | c.2327C>T,p.P776L | 1 | 8 | DHC_N1, stem, DLIC-interacting |
| Wang et al, 2018 |  | 103 | c.2327C>T,p.P776L | 1 | 8 | DHC_N1, stem, DLIC-interacting |
| Xing, 2020 | 57 (part) | 104 | c.751C>T, p.R251C | 1 | 3 | DHC_N1, stem |
| Zhang, 2014 | 58 | 105 | c.3538C>T,p.T1140M | 1 | 13 | stem |

*: "part" indicates that only parts of the family patient members were included in our study due to the fact that some patients lack detailed clinical information.
