# Supplementary material for: Expanding the Phenotypic and Genetic Spectrum of Neuromuscular Diseases Caused by DYNC1H1 Mutations
Source: Front Neurol. 2022 Jul 11;13:943324. doi: 10.3389/fneur.2022.943324 (PMC9309508; doi:10.3389/fneur.2022.943324)
Supplement: Supplementary Table 2 — Demographics and clinical characteristics of 105 patients with DYNC1H1 variants. [file Table_2.DOCX]

**Supplementary Table 2. Demographics and clinical characteristics of 105 patients with *DYNC1H1* variants**

| Reference | Family ID | Patient ID | Gender | Age (years) | Origin | Diagnosis | Onset age | Epilepsy | Delayed motor milestones | Atrophy of lower limbs | Weakness of lower limbs | Upper limb involvement | Trunk involvement | Abnormal tendon reflex | Ambulant | Gowers sign | Sensory abnormalities | Deformities | Intellectual disability | ADHD | Increased CK | Abnormal NCV | Abnormal brain MRI |
| --- | --- | --- | --- | --- | --- | --- | --- | --- | --- | --- | --- | --- | --- | --- | --- | --- | --- | --- | --- | --- | --- | --- | --- |
| Beecroft et al, 2017 | 1 (part*) | 1 | F | NA | Austrailia | SMALED1 | 2y | - | NA | + | + | - | + | + | + | - | - | + | - | - | - | + | - |
| Beecroft et al, 2017 |  | 2 | M | NA | Austrailia | SMALED1 | at birth | + | NA | - | + | - | - | - | + | - | - | + | - | + | - | - | - |
| Beecroft et al, 2017 | 2 | 3 | M | 14 | Austrailia | SMALED1 | at birth | - | + | + | + | - | - | + | + | - | - | + | - | - | NA | NA | NA |
| Beecroft et al, 2017 | 3 | 4 | M | 13 | Turkey | SMALED1 | at birth | - | + | + | + | + | - | + | + | + | - | + | - | - | NA | NA | NA |
| Beecroft et al, 2017 | 4 | 5 | M | 49 | Austrailia | SMALED1 | at birth | - | - | + | + | - | - | + | - | - | - | + | - | - | NA | + | NA |
| Viollet et al, 2020 | 5 (part) | 6 | M | 4 | France | SMALED1 | NA | NA | NA | + | + | NA | NA | + | + | NA | NA | + | NA | NA | NA | - | NA |
| Viollet et al, 2020 |  | 7 | F | 17 | France | SMALED1 | NA | NA | NA | NA | + | NA | NA | - | + | NA | NA | + | NA | NA | NA | - | NA |
| Viollet et al, 2020 |  | 8 | F | 15 | France | SMALED1 | NA | NA | NA | NA | + | NA | NA | + | + | NA | NA | + | NA | NA | NA | - | NA |
| Viollet et al, 2020 |  | 9 | M | 17 | France | SMALED1 | NA | NA | NA | + | + | NA | NA | + | + | NA | NA | + | NA | NA | NA | - | NA |
| Viollet et al, 2020 |  | 10 | F | 16 | France | SMALED1 | NA | - | - | + | + | - | - | + | + | + | - | + | - | - | NA | - | NA |
| Tsurusaki et al, 2012 | 6 | 11 | F | 18 | Japan | SMALED1 | infantile | - | + | + | + | - | - | + | + | + | - | - | - | - | - | - | - |
| Tsurusaki et al, 2012 |  | 12 | M | 12 | Japan | SMALED1 | 0.65y | - | + | + | + | - | - | - | + | - | - | - | - | - | - | - | - |
| Tsurusaki et al, 2012 |  | 13 | F | 50 | Japan | SMALED1 | NA | - | - | + | - | - | - | - | + | - | - | - | - | - | NA | NA | NA |
| Harms et al, 2012 | 7 (part) | 14 | NA | 74 | US | SMALED1 | infantile | - | - | + | + | + | - | + | + | NA | NA | + | - | - | NA | + | NA |
| Harms et al, 2012 | 8 | 15 | F | 3.5 | US | SMALED1 | infantile | - | + | - | + | - | - | NA | + | NA | - | + | + | - | NA | NA | NA |
| Niu et al, 2015 | 9 | 16 | M | 7 | China | SMALED1 | infantile | - | + | + | + | - | - | NA | + | - | - | NA | - | - | - | NA | - |
| Niu et al, 2015 |  | 17 | F | NA | China | SMALED1 | NA | - | NA | + | + | NA | NA | NA | + | + | - | NA | - | - | NA | NA | NA |
| Niu et al, 2015 |  | 18 | M | NA | China | SMALED1 | NA | - | NA | + | + | NA | NA | NA | + | + | - | NA | - | - | NA | NA | NA |
| Scoto et al, 2015 | 10 | 19 | NA | 40s | UK | SMALED1 | adult | - | - | + | + | - | - | + | + | - | - | + | - | - | NA | NA | NA |
| Scoto et al, 2015 |  | 20 | NA | 9.5 | UK | SMALED1 | at birth | - | - | + | + | + | - | + | - | - | - | + | + | + | NA | NA | NA |
| Scoto et al, 2015 | 11 | 21 | NA | 10 | UK | SMALED1 | at birth | - | - | + | + | + | - | + | - | - | - | + | + | + | NA | NA | NA |
| Scoto et al, 2015 | 12 | 22 | NA | 5 | UK | SMALED1 | <2y | - | + | + | + | - | - | + | + | - | - | + | + | + | NA | NA | NA |
| Scoto et al, 2015 |  | 23 | NA | 9 | UK | SMALED1 | <2y | - | + | + | + | + | - | + | + | - | - | + | + | + | NA | NA | NA |
| Scoto et al, 2015 | 13 | 24 | NA | 40s | UK | SMALED1 | childhood | - | - | + | + | - | - | NA | + | - | - | NA | - | - | NA | NA | NA |
| Scoto et al, 2015 |  | 25 | NA | 16 | UK | SMALED1 | at birth | - | - | + | + | - | - | + | + | - | - | + | - | - | NA | NA | NA |
| Scoto et al, 2015 | 14 | 26 | NA | 8 | UK | SMALED1 | at birth | - | - | + | + | - | - | + | + | - | - | + | - | - | NA | NA | NA |
| Scoto et al, 2015 | 15 | 27 | NA | 5 | UK | SMALED1 | at birth | - | - | + | + | - | - | + | + | - | - | + | - | - | NA | NA | NA |
| Scoto et al, 2015 | 16 (part) | 28 | NA | 3.5 | UK | SMALED1 | at birth | - | - | + | + | - | - | + | + | - | - | + | - | - | NA | NA | NA |
| Scoto et al, 2015 | 17 | 29 | NA | 2.5 | UK | SMALED1 | at birth | + | - | + | + | - | - | + | - | - | - | + | + | - | NA | NA | NA |
| Scoto et al, 2015 | 18 | 30 | NA | 3 | UK | SMALED1 | at birth | - | - | + | + | + | - | NA | - | - | - | + | NA | NA | NA | NA | NA |
| Scoto et al, 2015 | 19 | 31 | NA | 80s | Spain | SMALED1 | late 40s | - | - | + | + | - | - | + | + | - | - | + | - | - | NA | NA | NA |
| Scoto et al, 2015 |  | 32 | NA | 50s | Spain | SMALED1 | childhood | - | - | - | + | - | - | + | + | - | - | + | - | - | NA | NA | NA |
| Scoto et al, 2015 |  | 33 | NA | 50s | Spain | SMALED1 | 20s | - | - | + | + | - | - | + | + | - | - | + | - | - | NA | NA | NA |
| Scoto et al, 2015 |  | 34 | NA | 22 | Spain | SMALED1 | 20s | - | - | - | - | - | - | + | + | - | - | + | - | + | NA | NA | NA |
| Scoto et al, 2015 |  | 35 | NA | 37 | Spain | SMALED1 | childhood | - | - | + | + | - | - | + | + | - | - | + | - | - | NA | NA | NA |
| Scoto et al, 2015 | 20 (part) | 36 | NA | 10 | US | SMALED1 | <2y | - | + | + | + | - | - | + | + | - | - | + | + | - | NA | NA | NA |
| Scoto et al, 2015 |  | 37 | NA | 9 | US | SMALED1 | <2y | + | + | + | + | - | - | + | + | - | - | + | + | - | NA | NA | NA |
| Scoto et al, 2015 | 21 | 38 | NA | 15 | US | SMALED1 | at birth | - | - | + | + | - | - | + | + | - | - | + | + | - | NA | NA | NA |
| Scoto et al, 2015 | 22 | 39 | NA | 56 | US | SMALED1 | 25y | - | - | - | + | - | - | + | + | - | - | - | - | - | NA | NA | NA |
| Scoto et al, 2015 |  | 40 | NA | 62 | US | SMALED1 | 32y | - | - | - | + | - | - | + | + | - | - | - | - | - | NA | NA | NA |
| Scoto et al, 2015 | 23 | 41 | NA | 1 | Sweden | SMALED1 | at birth | - | - | - | + | - | - | - | + | - | - | + | - | - | NA | NA | NA |
| Scoto et al, 2015 |  | 42 | NA | 30s | Sweden | SMALED1 | <1y | - | - | - | + | - | + | - | + | - | - | + | - | - | NA | NA | NA |
| Scoto et al, 2015 | 24 | 43 | NA | 11 | Sweden | SMALED1 | <1y | - | + | + | + | - | - | + | + | - | - | + | - | - | NA | NA | NA |
| Scoto et al, 2015 |  | 44 | NA | 45 | Sweden | SMALED1 | childhood | - | - | + | + | - | - | + | + | - | - | + | - | - | NA | NA | NA |
| Scoto et al, 2015 | 25 | 45 | NA | 5 | Netherlands | SMALED1 | childhood | - | - | + | + | - | - | + | + | - | - | + | + | - | NA | NA | NA |
| Scoto et al, 2015 |  | 46 | NA | 40s | Netherlands | SMALED1 | 20s | - | - | + | + | - | - | + | + | - | - | + | + | - | NA | NA | NA |
| Punetha et al, 2015 | 26 | 47 | F | 3.6 | US | SMALED1 | at birth | + | + | + | + | - | - | + | + | NA | - | + | - | - | - | - | NA |
| Chen et al, 2017 | 27 | 48 | F | 1.3 | China | SMALED1 | at birth | - | + | NA | + | - | - | + | - | NA | - | + | NA | NA | - | + | + |
| Chan et al, 2018 | 28 | 49 | F | 15 | Middle East | SMALED1 | 1.7y | - | + | + | + | + | - | NA | - | - | - | + | + | - | - | - | + |
| Chan et al, 2018 | 29 | 50 | F | 18 | China | SMALED1 | 2y | - | + | + | + | + | - | + | + | + | - | + | - | + | + | - | + |
| Chan et al, 2018 | 30 | 51 | M | 12 | China | SMALED1 | 2y | - | + | + | + | + | - | + | + | + | - | + | - | + | + | - | + |
| Chan et al, 2018 | 31 | 52 | M | 21 | Europe | SMALED1 | 3y | - | + | + | + | + | - | + | + | - | - | + | + | + | - | - | + |
| Das et al, 2018 | 32 | 53 | F | 82 | UK | SMALED1 | at birth | - | NA | + | + | - | - | NA | + | NA | NA | - | - | - | - | - | NA |
| Das et al, 2018 |  | 54 | M | 60 | UK | SMALED1 | at birth | + | NA | + | + | - | - | NA | + | NA | NA | - | + | - | - | - | NA |
| Das et al, 2018 |  | 55 | M | 59 | UK | SMALED1 | at birth | - | NA | - | + | - | - | NA | + | NA | NA | + | - | - | - | - | NA |
| Das et al, 2018 |  | 56 | M | 58 | UK | SMALED1 | at birth | - | NA | + | + | - | - | + | + | NA | - | + | - | - | - | - | NA |
| Das et al, 2018 |  | 57 | M | 32 | UK | SMALED1 | at birth | - | NA | + | + | - | - | + | + | NA | - | + | - | - | - | - | NA |
| Das et al, 2018 |  | 58 | M | 30 | UK | SMALED1 | at birth | - | NA | + | + | - | - | + | + | NA | - | + | - | - | - | - | NA |
| Weedon et al, 2011 | 33 (part) | 59 | M | NA | UK | CMT2O | infantile | - | + | + | + | + | - | - | + | NA | + | + | - | - | NA | - | NA |
| Weedon et al, 2011 |  | 60 | M | NA | UK | CMT2O | at birth | - | - | + | + | - | - | - | + | NA | + | + | - | - | NA | - | NA |
| Weedon et al, 2011 |  | 61 | M | NA | UK | CMT2O | infantile | - | + | + | + | - | - | - | + | NA | + | + | - | - | NA | - | NA |
| Weedon et al, 2011 |  | 62 | M | NA | UK | CMT2O | 11y | - | - | - | + | - | - | + | + | NA | + | + | - | - | NA | - | NA |
| Weedon et al, 2011 |  | 63 | M | NA | UK | CMT2O | 14y | - | - | + | + | + | - | + | + | NA | + | + | - | - | NA | - | NA |
| Weedon et al, 2011 |  | 64 | M | NA | UK | CMT2O | at birth | - | + | + | + | - | - | - | + | NA | - | + | - | - | NA | - | NA |
| Weedon et al, 2011 |  | 65 | M | NA | UK | CMT2O | infantile | - | + | + | + | - | - | + | + | NA | - | + | - | - | NA | - | NA |
| Weedon et al, 2011 |  | 66 | M | NA | UK | CMT2O | early childhood | - | - | + | + | - | - | - | + | NA | - | - | - | - | NA | - | NA |
| Weedon et al, 2011 |  | 67 | M | NA | UK | CMT2O | infantile | - | + | + | + | - | + | + | + | NA | + | + | + | - | NA | - | NA |
| Weedon et al, 2011 |  | 68 | M | NA | UK | CMT2O | infantile | - | + | + | + | - | - | + | + | NA | - | + | + | - | NA | - | NA |
| Weedon et al, 2011 |  | 69 | M | NA | UK | CMT2O | early childhood | - | + | - | + | - | - | - | + | NA | - | - | + | - | NA | - | NA |
| Weedon et al, 2011 |  | 70 | M | NA | UK | CMT2O | infantile | - | + | + | + | - | - | + | + | NA | + | + | + | - | NA | - | NA |
| Weedon et al, 2011 |  | 71 | M | NA | UK | CMT2O | infantile | - | + | - | + | - | - | + | + | NA | - | + | - | - | NA | - | NA |
| Argente-Escrig et al, 2020 | 34 | 72 | NA | 18 | Austrailia | SMALED1 | at birth | - | + | NA | NA | NA | NA | NA | - | NA | NA | + | + | - | NA | + | NA |
| Argente-Escrig et al, 2020 | 35 | 73 | NA | 16 | Spain | SMALED1 | <1y | - | - | NA | NA | NA | NA | NA | + | NA | NA | + | + | - | NA | - | - |
| Peeters et al, 2015 | 36 (part) | 74 | F | 52 | Austrailia | CMT2O | 1y | - | + | + | + | + | - | + | - | NA | + | + | - | - | NA | - | NA |
| Peeters et al, 2015 |  | 75 | F | 25 | Austrailia | CMT2O | 1-2y | - | + | + | + | - | - | + | + | NA | + | + | - | - | NA | - | NA |
| Peeters et al, 2015 | 37 | 76 | F | 7 | Bulgaria | SMALED1 | infantile | - | + | + | + | - | - | + | - | NA | - | + | - | - | - | - | - |
| Amabile et al, 2020 | 38 | 77 | F | 4 | US | SMALED1 | at birth | + | + | NA | + | + | + | NA | + | NA | - | + | + | - | - | NA | + |
| Amabile et al, 2020 | 39 | 78 | F | 10.8 | US | SMALED1 | 1.5y | - | - | + | + | + | - | NA | - | NA | - | + | - | - | NA | NA | NA |
| Becker et al, 2020 | 40 | 79 | M | NA | Germany | Others | infantile | - | + | + | + | + | - | + | + | + | NA | + | + | + | NA | - | - |
| Becker et al, 2020 | 41 | 80 | M | NA | Germany | Others | infantile | + | + | + | + | - | - | + | + | - | NA | + | + | - | NA | + | + |
| Becker et al, 2020 | 42 | 81 | F | NA | Germany | Others | infantile | - | + | + | + | - | - | + | + | - | NA | + | + | - | NA | - | + |
| Becker et al, 2020 | 43 | 82 | F | NA | Germany | Others | infantile | + | + | + | + | - | - | - | + | + | NA | + | + | - | NA | NA | + |
| Becker et al, 2020 | 44 | 83 | M | NA | Germany | Others | infantile | - | + | + | + | - | - | + | NA | - | NA | + | + | - | NA | + | + |
| Becker et al, 2020 | 45 | 84 | M | NA | Germany | Others | infantile | - | + | + | + | + | - | + | + | - | NA | + | - | - | NA | NA | - |
| Becker et al, 2020 | 46 | 85 | M | NA | Germany | Others | infantile | - | + | + | + | + | - | + | NA | - | NA | + | - | + | NA | NA | + |
| Becker et al, 2020 | 47 | 86 | M | NA | Germany | Others | infantile | + | + | + | + | - | - | + | + | - | NA | + | + | - | NA | + | + |
| Becker et al, 2020 | 48 | 87 | M | NA | Germany | Others | infantile | - | + | - | - | - | - | - | + | - | NA | + | + | + | NA | NA | + |
| Becker et al, 2020 | 49 | 88 | F | NA | Germany | Others | infantile | + | - | - | - | - | - | - | + | - | NA | + | + | - | NA | NA | + |
| Fiorillo et al, 2014 | 50 | 89 | M | 19 | Italy | Others | at birth | - | - | + | + | - | - | NA | + | NA | - | + | + | - | NA | - | + |
| Fiorillo et al, 2014 | 51 | 90 | M | 9 | Italy | Others | at birth | - | + | + | + | - | + | + | - | NA | - | + | + | + | NA | - | + |
| Strickland et al, 2015 | 52 | 91 | M | NA | Austria | SMALED1 | at birth | - | + | + | + | - | - | + | + | NA | - | + | NA | NA | NA | + | NA |
| Strickland et al, 2015 |  | 92 | NA | NA | Austria | SMALED1 | at birth | - | + | + | + | - | - | + | + | NA | - | + | NA | NA | NA | - | NA |
| Strickland et al, 2015 |  | 93 | NA | NA | Austria | SMALED1 | infantile | - | + | + | + | - | - | + | + | NA | - | + | NA | NA | NA | - | NA |
| Strickland et al, 2015 | 53 | 94 | M | NA | Spain | SMALED1 | at birth | - | + | + | + | - | - | + | + | NA | - | + | NA | NA | - | NA | NA |
| Singh et al, 2015 | 54 | 95 | M | NA | UK | SMALED1 | at birth | + | NA | NA | NA | - | - | + | NA | NA | NA | + | NA | NA | NA | + | + |
| Ding et al, 2017 | 55 | 96 | F | 24 | China | SMALED1 | at birth | - | + | + | + | - | - | + | + | NA | - | + | - | - | - | - | - |
| Ding et al, 2017 |  | 97 | F | 24 | China | SMALED1 | at birth | - | + | + | + | - | - | + | + | NA | - | + | - | - | - | - | - |
| Wang et al, 2018 | 56 (part) | 98 | M | 49 | China | SMALED1 | NA | - | + | + | + | - | - | + | + | NA | - | + | - | - | NA | NA | NA |
| Wang et al, 2018 |  | 99 | F | 45 | China | SMALED1 | NA | - | + | + | + | - | - | + | + | NA | - | + | - | - | NA | NA | NA |
| Wang et al, 2018 |  | 100 | F | 39 | China | SMALED1 | NA | - | + | + | + | - | - | + | + | NA | - | + | - | - | NA | NA | NA |
| Wang et al, 2018 |  | 101 | M | 18 | China | SMALED1 | NA | - | + | - | + | - | - | + | + | NA | - | + | - | - | NA | NA | NA |
| Wang et al, 2018 |  | 102 | M | 17 | China | SMALED1 | NA | - | + | + | + | - | - | + | + | NA | - | + | - | - | + | - | - |
| Wang et al, 2018 |  | 103 | F | 7 | China | SMALED1 | NA | - | + | + | + | - | - | + | - | NA | - | + | - | - | NA | NA | NA |
| Xing, 2020 | 57 (part) | 104 | F | 9 | China | SMALED1 | 1y | - | - | + | + | - | - | + | + | NA | + | + | - | - | - | - | + |
| Zhang, 2014 | 58 | 105 | F | 3 | China | CMT2O | NA | - | + | + | + | - | - | + | + | NA | - | - | - | - | NA | - | - |

*: "part" indicates that only parts of the family patient members were included in our study due to the fact that some patients lack detailed clinical information.

Abbreviations: CMT2O, Charcot-Marie-Tooth disease type 2O; F, female; M, male; NA: Not applicable; SMALED1: Spinal muscular atrophy, lower extremity-predominant 1.
